# Dual-Stream SPP-CNN for High-Precision sEMG Gesture Recognition in Human–Machine Interfaces

**DOI:** 10.3390/biomimetics11070508

**Published:** 2026-07-19

**Authors:** Zebin Li, Gang Zhang, Lifu Gao, Wenming Wang, Wei Lu, Guocai Liu, Jinzhong Zhang

**Affiliations:** 1 Intelligent Control and Robotics Research Center, West Anhui University, Lu’an 237012, China; zhanggang@wxc.edu.cn (G.Z.); 43000003@wxc.edu.cn (W.W.); 42000032@wxc.edu.cn (G.L.); 42000023@wxc.edu.cn (J.Z.); 2 Institute of Intelligent Machines, Hefei Institutes of Physical Science, Chinese Academy of Sciences, Hefei 230031, China; lifugao@iim.ac.cn; 3 Department of Science Island, University of Science and Technology of China, Hefei 230031, China; 4 School of Management, Fujian University of Technology, Fuzhou 350118, China; lw9296@mail.ustc.edu.cn

**Keywords:** muscle–computer interface, surface electromyography, gesture recognition, CWT, GADF, dual-stream convolutional neural network

## Abstract

Surface electromyography (sEMG) signals directly reflect movement intention and are therefore promising for natural human–machine interaction. However, their inherent non-stationarity and high inter-subject variability remain major obstacles to robust feature extraction and model generalization. To address these challenges, this study proposes a dual-stream spatial pyramid pooling convolutional neural network (DSSCNN). In this framework, one-dimensional sEMG segments are transformed into two complementary image representations, continuous wavelet transform (CWT) spectrograms and Gramian angular difference field (GADF) images, forming a dual-channel input that jointly preserves time–frequency dynamics and temporal correlation structures. A dual-stream convolutional architecture then extracts discriminative features from each modality, after which a spatial pyramid pooling (SPP) layer aggregates multi-scale representations, enhancing the network’s capacity to capture robust spatiotemporal patterns. Extensive experiments demonstrate that DSSCNN achieves an average gesture recognition accuracy of 97.88% with low inter-subject variance under intra-subject random split, and 96.59% under leave-one-subject-out (LOSO) protocol. The practical viability of the proposed approach is further validated through real-time control of an unmanned ground vehicle (UGV). These results not only indicate that the dual-stream framework combined with SPP layer provides an effective strategy for high-precision sEMG-based gesture recognition but also provides a promising technical pathway toward next-generation natural human–machine interaction.

## 1. Introduction

As a natural and intuitive medium of communication, the hand offers significant advantages in expressing emotions and conveying intentions, making it an ideal interface for human–computer interaction (HCI) [1,2,3,4,5]. Surface electromyography (sEMG) directly reflects forearm muscle activity and has therefore attracted substantial attention in intelligent recognition systems. Muscle–computer interfaces (MCIs) based on sEMG have been successfully applied in fields such as robot control, prosthetic operation, virtual reality, and driver assistance [2], where accurate gesture recognition plays a crucial role in daily life and industrial production. However, the gesture diversity and environmental complexity still reduce prediction accuracy, making high-precision, high-efficiency sEMG gesture recognition a persistent research challenge [3].

Surface electromyography signals originate from the temporal and spatial superposition of motor unit action potentials generated by multiple muscle fibers and can be recorded non-invasively at the skin surface [4]. Because these signals can be detected prior to the onset of muscle contraction and effectively characterize both muscle activation levels and movement intention, they serve as an ideal control source for rapid and reliable HCI. However, when sEMG is employed as a control source under non-ideal conditions, its robustness and recognition accuracy frequently suffer substantial declines, and numerous practical challenges remain [5].

Surface electromyography gesture recognition has become an important research direction in HCI [6]. The core objective is to identify muscle contraction patterns and movement intentions from sEMG signals and map them to predefined gestures via supervised learning. The technical pipeline comprises three stages: signal acquisition, preprocessing, and classification. Sparse electrode systems facilitate data collection but yield limited information, whereas dense two-dimensional arrays capture richer spatiotemporal variations at the cost of increased processing complexity. Preprocessing filters noise and extracts valid muscle activity segments, typically involving bandpass filtering (20–500 Hz), notch filtering (50 Hz), and signal normalization. Classification approaches comprise traditional machine learning and deep learning. Traditional classifiers, such as random forests (RFs) [7], multilayer perceptrons (MLPs) [8], and support vector machines (SVMs) [9], rely heavily on manually designed time-domain, frequency-domain, or time–frequency features. Their performance depends on feature quality and the classifier. When such features fail to capture signal–action relationships, recognition performance declines substantially with increasing gesture categories, and cross-subject generalization is also affected. Consequently, models enabling automatic sEMG feature learning are crucial for improving recognition accuracy.

Deep learning, particularly convolutional neural networks (CNNs), has demonstrated strong capabilities in automatic feature extraction from complex surface sEMG signals, making it increasingly attractive for industrial automation, rehabilitation, and virtual reality [2,10]. Unlike traditional methods that rely on manually designed features, CNNs can hierarchically learn representative signal patterns through convolution and pooling operations, enabling effective modeling of high-dimensional, nonlinear sEMG data. Previous studies have attempted to enhance gesture recognition by manually extracting multi-dimensional features from raw sEMG signals [1,9]. Although these approaches can improve accuracy, they add computational cost and do not fully exploit end-to-end feature learning. CNN-based methods partly address this problem. For example, Kim et al. [11] achieved over 90% recognition accuracy by applying CNNs to raw EMG signals, and Atzori et al. [12] demonstrated superior performance using a two-dimensional matrix formed by time windows covering all available electrode measurements as CNN input. Nevertheless, these methods may insufficiently capture temporal correlations and time–frequency details, which are crucial for fully exploiting the dynamic characteristics of sEMG signals.

Transforming one-dimensional sEMG time series into two-dimensional representations creates new opportunities for end-to-end feature learning. Geng et al. [13] constructed instantaneous sEMG images from high-density electrode arrays and used them as CNN inputs, achieving superior performance over conventional classifiers across multiple datasets. Wei et al. [2] further decomposed instantaneous images into equal-sized patches and processed them with a multi-stream CNN, achieving a maximum gesture recognition accuracy of 85.0% on the NinaPro database with a 200 ms time window. However, directly converting EMG time series into instantaneous amplitude images inevitably discards temporal correlation information and provides incomplete time–frequency descriptions. To address this limitation, time–frequency analysis and geometric coding methods, including CWT, short-time Fourier transform (STFT), and Gramian angular field (GAF) encoding, have attracted widespread attention [14,15,16,17]. For instance, Shafaa et al. [14] employed CWT to transform EMG signals into time–frequency images, extracted features via CNN, and subsequently classified them using least-squares support vector machines (LS-SVM), achieving 95.33% accuracy. Jo et al. [15] systematically compared STFT, CWT, and scale-averaged wavelet transform (SAWT) as preprocessing strategies and subsequently applied a convolutional recurrent neural network (CRNN) with superior performance. Similarly, Fan et al. [17] leveraged GAF transformation to construct representations capable of encoding both instantaneous signal values and temporal dependencies, which were processed by CNN-based models, yielding competitive recognition outcomes.

CNNs have demonstrated unique advantages in deep learning classification tasks; however, they exhibit inherent limitations in modeling global contextual information. This deficiency becomes notably challenging for tasks that require distinguishing between similar yet distinct motor intentions. To address this challenge, multi-stream CNN (MSCNN) architectures have emerged as a promising solution for enhancing recognition accuracy and robustness [2,18,19]. Wei et al. [2] demonstrated that the MSCNN architecture outperforms a single-stream CNN (SSCNN) in sEMG-based gesture recognition by decomposing raw sEMG images into data streams of equal size. Yang et al. [18] designed a multi-stream residual network (MResLSTM) that extracts global and deep spatiotemporal features, achieving high-precision dynamic sEMG gesture recognition. Similarly, Xu et al. [19] proposed a dual-stream CNN (DSCNN) that achieves superior performance in gesture recognition tasks through dual-modal feature fusion of sEMG energy kernel phase portrait and IMU amplitude image.

In summary, despite recent advancements in deep learning, existing models still face challenges in achieving high recognition accuracy, effective feature extraction, and real-time performance. In particular, CNN-based sEMG gesture recognition has not fully exploited the information potential contained in short-window sEMG signals. To overcome these limitations, this paper proposes a dual-stream spatial pyramid pooling CNN (DSSCNN), a novel dual-stream convolutional architecture. The main contributions of this work are as follows:

(1) SEMG signals are converted into complementary dual-channel images via CWT and GADF, preserving time–frequency details and temporal correlations.
(2) The classifier incorporates an SPP layer that aggregates multi-scale features, thereby strengthening discriminative capacity and reducing the number of learnable parameters.
(3) The dual-stream CNN architecture employs a divide-and-conquer strategy to automatically learn features from CWT and GADF images, eliminating manual feature engineering and improving recognition performance.
(4) The proposed framework is evaluated on both multi-subject gesture datasets and real-time UGV control experiments, confirming its robustness and practical value.

## 2. Materials and Methods

### 2.1. Experimental Devices and Procedures

**Figure 1:**
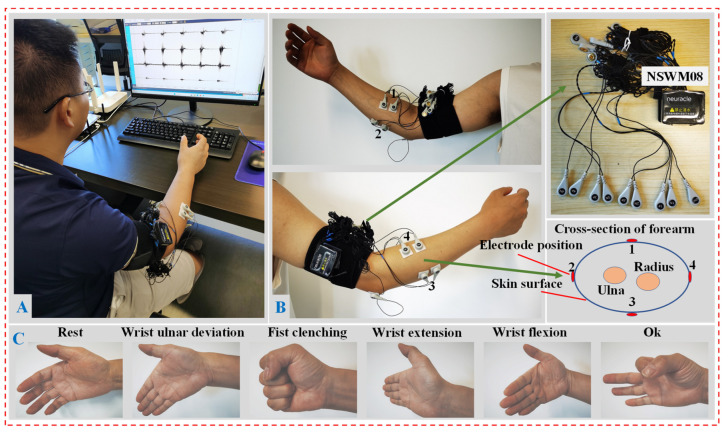
Experimental setup and protocol for sEMG-based gesture recognition. (**A**) Integrated real-time data acquisition platform; (**B**) spatial layout of the multichannel hardware system, showing the placement of four electrodes on the forearm, the NSWM08 acquisition module, and a schematic cross-section of the forearm; and (**C**) gesture labels used in the experimental paradigm, including rest, wrist ulnar deviation, fist clenching, wrist extension, wrist flexion, and OK gesture.

To acquire sEMG signals, the commercial wireless portable NSWM08 bipolar sEMG acquisition system (Neuracle Technology Co., Ltd., Changzhou, China) was adopted with a 1 kHz sampling rate, as shown in Figure 1. The device provides eight acquisition channels, of which four were utilized in this study. The electrodes were placed on the proximal one-third of the forearm measured from the elbow joint toward the wrist. To reduce dependence on a single muscle belly and capture circumferential forearm activation, the four bipolar channels were arranged around the forearm at approximately equal angular intervals; the inter-electrode distance within each channel was maintained at 20 mm. The skin was cleaned before attachment, and the same anatomical rule was used for all participants. This study did not include separate experiments with abnormal electrode placements; therefore, the present robustness results should be interpreted as robustness under standardized circumferential placement conditions, rather than under arbitrary sensor displacement. The experimental setup and electrode placement configuration are illustrated in Figure 1.

Drawing upon conventional hand gestures for UGV control, this study selected five basic movements: forward, backward, right turn, left turn, and stop. As shown in Figure 1C, the corresponding hand gesture commands mapped to these UGV motions included wrist ulnar deviation, fist clenching, wrist extension, wrist flexion, and the “OK” gesture. The gesture recognition experiment recruited nine healthy subjects (aged 22–24 years; six males and three females). All participants were in good health with no history of musculoskeletal or neurological disorders. During the experiment, sEMG data were collected while participants executed the five distinct gestures. Guided by prompt instructions, participants completed each gesture cycle within a 3 s window, which consisted of a 1 s sustained gesture followed by a 2 s relaxation period prior to the subsequent movement. The experimental protocol was approved by the Ethics Review Committee of West Anhui University (protocol code No. 202512005; approval date: 19 December 2025). Before participation, all participants provided written informed consent after being fully informed about the purpose, procedures, low-risk/non-invasive nature of the recording process, voluntary participation, confidentiality protections, and the right to withdraw from the study at any time without penalty. They subsequently completed a health history questionnaire and signed a written informed consent form.

### 2.2. Signal Processing and Dynamic Hand Gesture Segmentation

**Figure 2:**
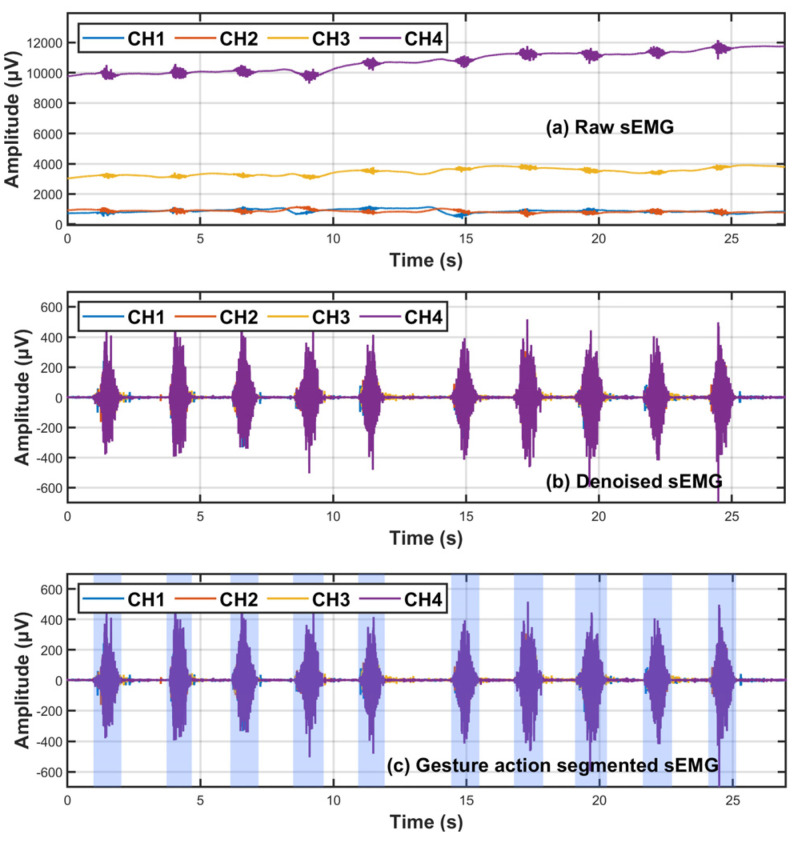
sEMG signal preprocessing: (**a**) raw sEMG, (**b**) denoised sEMG with a 20–500 Hz Butterworth filter, and (**c**) gesture action segmented sEMG based on a sliding window root mean square (RMS) energy detector.

Raw sEMG signals typically suffer from substantial background noise; therefore, robust preprocessing is indispensable for subsequent feature extraction [20,21]. A segment of raw sEMG data from the experiment is illustrated in Figure 2a. In sEMG-based motion recognition and classification, preprocessing exerts a profound influence on the final accuracy. The processing workflow comprises three consecutive stages: digital filtering, Z-score standardization, and sliding-window-based gesture segmentation. To suppress noise, the raw signals were first passed through a fourth-order Butterworth bandpass filter (20–500 Hz), combined with a 50 Hz notch filter to eliminate power line interference. The filtering strategy effectively preserved the dominant energy components of the target signal. Given that filtered sEMG amplitudes are inherently weak and susceptible to inter-subject variability, Z-score standardization was applied to rescale the data to a standardized distribution. The mathematical expression is as follows:

(1) $$z=\frac{x-\mu }{\sigma }$$

where μ and σ denote the mean and standard deviation of a given electrode channel data, respectively. This channel-wise standardization preserved the intrinsic signal distribution within each channel while mitigating the impact of outliers, thereby clustering the data into a highly discriminative subspace. The resulting preprocessed sEMG signals are depicted in Figure 2b.

Due to inherent inter-subject variations in the onset and offset times of hand gestures, precisely isolating active muscle contractions from baseline resting states is imperative [22]. To address this challenge, the specific detection pipeline comprises the following stages: First, the raw multichannel sEMG signals undergo full-wave rectification and low-pass filtering to extract smooth, non-negative signal envelopes. Second, these individual envelopes are fused by calculating their cross-channel average. Subsequently, a sliding window root mean square (RMS) energy detector tracks the time-domain intensity of the fused envelope. Finally, gesture activity segments are isolated by applying an empirical energy threshold, thereby isolating intervals that exceed this baseline. The sliding window RMS energy is mathematically defined as

(2) $$E_{RMS}\left[k\right]=\sqrt{\frac{1}{N_{w}}\sum_{m=0}^{N_{w}-1}\;x_{\mathrm{fused}}^{2}\left[k\cdot N_{s}+m\right]}$$

where $E_{\mathrm{RMS}}\left[k\right]$ represents the RMS energy of the k-th window, $x_{\mathrm{fused}}$ is the fused sEMG envelope signal, and $N_{w}$ denotes the window length. This length is governed by the temporal window duration ($T_{w}$) and the sampling frequency ($f_{s}$), as expressed in Equation (3):

(3) $$N_{w}=T_{w}\times f_{s}$$

where $N_{s}$ is the window step size, defined as the displacement between the onset of consecutive windows. It is calculated from the temporal step size ($T_{s}$) and the sampling frequency ($f_{s}$), as shown in Equation (4):

(4) $$N_{S}=T_{s}\times f_{s}$$

In this study, the temporal window size ($T_{w}$) and step size ($T_{s}$) were configured to 150 ms and 50 ms, respectively.

As illustrated in Figure 2c, the segmented active hand gestures exhibit variable durations. To minimize computational latency and meet real-time control constraints, a sliding window was applied to uniformly segment the active sEMG sequences, thereby standardizing the input dimensions for the deep learning network. The window width was configured to 256 data points (corresponding to 256 ms at the 1 kHz sampling rate), with a step size of 128 data points (128 ms).

### 2.3. Continuous Wavelet Transform

To accommodate the two-dimensional input topology of CNN classifiers, mapping one-dimensional sEMG time series signals into the 2D domain establishes a structural alignment that preserves localized energy patterns. Given that sEMG signals are inherently stochastic and non-stationary, CWT, as a robust mathematical approach, projects time-domain signals into the time–frequency domain via localized basis functions [23,24]. Because multi-scale spectral representations effectively capture non-stationary transient dynamics of muscle contractions, the CWT was employed to transform multichannel sEMG signals into two-dimensional scalograms. Unlike the STFT, which is constrained by a fixed time–frequency resolution trade-off, the CWT employs an adaptive time–frequency windowing mechanism. Consequently, the target signal is decomposed onto a basis generated through the translation and scaling of a localized mother wavelet. The CWT is formally defined as follows [23,25]:

(5) $$W_{f}\left(a,b\right)=\frac{1}{\sqrt{\left|a\right|}}\int_{-\infty }^{+\infty }\;s\left(t\right)\Psi^{*}\left(\frac{t-b}{a}\right)dt$$

where $W_{f}\left(a,b\right)$ represents the resultant wavelet coefficients, $s\left(t\right)$ is the input sEMG signal, $\Psi \left(t\right)$ denotes the mother wavelet, and * signifies its complex conjugate. The parameter a scales the mother wavelet (inversely proportional to frequency), while b dictates its temporal translation (displacement over time).

**Figure 3:**
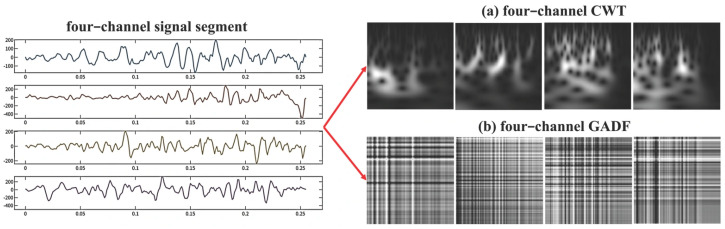
Images converted from four-channel signal segments: (**a**) four-channel CWT spectrograms and (**b**) four-channel GADF temporal correlation maps.

To isolate instantaneous amplitude dynamics and optimize transient feature capture, this study selected an analytic Morlet wavelet configured with 12 voices per octave, owing to its superior simultaneous temporal and spectral localization. By bypassing the computationally heavy and error-prone manual feature engineering in conventional methodologies, the proposed end-to-end framework simplifies feature representation learning [26]. Specifically, the multichannel signal segment within each sliding window was transformed into a composite matrix containing four distinct two-dimensional scalograms, as illustrated in Figure 3.

### 2.4. Gramian Angular Difference Field

To preserve temporal dependencies while extracting spatialized structural features, the Gramian angular field (GAF) is introduced to transform one-dimensional sEMG time series into two-dimensional images. By constructing a localized polar coordinate matrix, this technique explicitly retains the absolute temporal dependencies embedded within the biological sequences while establishing an end-to-end mapping from non-stationary physiological states to high-fidelity image representations [5,25].

Given a discrete, single-channel sEMG segment denoted by the sequence $X=\{x_{1},x_{2},\ldots ,x_{m}\}$, $x_{i}$ represents the continuous voltage amplitude captured at the i-th sampling temporal instance. To satisfy the angular constraints of the inverse cosine transform, the raw sequence must first be strictly restricted to a standardized interval of $\left[-1,\;1\right]$ via Equation (6).

(6) $${\tilde{x}}_{i}=\frac{\left(x_{i}-max\left(X\right)\right)+\left(x_{i}-min\left(X\right)\right)}{max\left(X\right)-min\left(X\right)}$$

where $\tilde{x_{i}}$ denotes the scaled amplitude. This scaling preserves the inherent dynamic range of the original sEMG while normalizing the data distribution before the polar transform, thereby reducing the variance caused by magnitude differences.

Subsequently, polar coordinate mapping is performed using Equation (7).

(7) $$\begin{array}{c} \left\{\begin{array}{c} \phi =arccos{\tilde{x}}_{i};-1\leq {\tilde{x}}_{i}\leq 1,{\tilde{x}}_{i}\in \tilde{X}\\ r=\frac{t_{i}}{N};t_{i}\in N \end{array}\right. \end{array}$$

where $t_{i}$ represents the timestamp, and r denotes the polar coordinate radius. By seamlessly integrating temporal sequences with the angular parameter ϕ constrained within the domain $\left[0,\pi \right]$, this coordinate mapping ensures that each discrete data point in the temporal sequence is assigned a unique and defined spatial location on a two-dimensional polar coordinate plane.

The GAF comprises distinct variants: the Gramian angular summation field (GASF) and the Gramian angular difference field (GADF) [27]. Although both variants leverage triangular operations within spatial intervals to capture temporal correlations, they exhibit significant differences in terms of numerical texture and feature extraction performance. Specifically, GADF displays superior edge sharpness and more distinct color contrast distributions. Consequently, it is capable of more effectively capturing the localized relative variations and transient fluctuations between adjacent samples of sEMG signals. The GADF matrix operator is formulated as follows [28]:

(8) $$GADF=\left[\begin{array}{cccc} \sin\left(\phi_{1}-\phi_{1}\right) & \sin\left(\phi_{1}-\phi_{2}\right) & \ldots & \sin\left(\phi_{1}-\phi_{n}\right)\\ \sin\left(\phi_{2}-\phi_{1}\right) & \sin\left(\phi_{2}-\phi_{2}\right) & \ldots & \sin\left(\phi_{2}-\phi_{n}\right)\\ \ldots & \ldots & \ldots & \ldots \\ \sin\left(\phi_{n}-\phi_{1}\right) & \sin\left(\phi_{n}-\phi_{2}\right) & \ldots & \sin\left(\phi_{n}-\phi_{n}\right) \end{array}\right]$$

The GADF transform preserves the temporal correlation structure of the original signal. In this study, four-channel sEMG segments were mapped into two-dimensional GADF images, as illustrated in Figure 3. Together with the CWT spectrograms, the GADF images provide complementary representations for the subsequent dual-stream CNN.

### 2.5. Network Structure of DSSCNN

**Figure 4:**
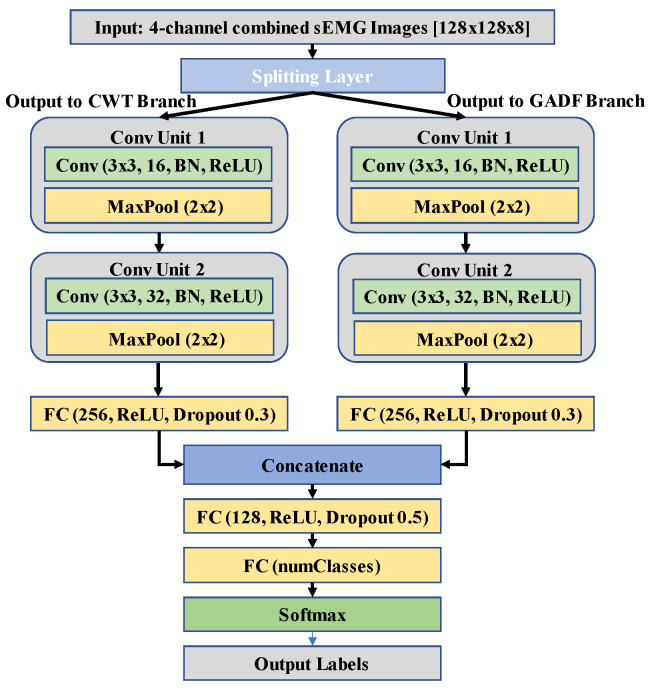
Baseline DSCNN architecture (without SPP) for multimodal feature extraction.

As illustrated in Figure 4, the proposed dual-stream CNN (DSCNN) serves as the backbone network prior to the addition of SPP layers; it primarily comprises convolutional layers, pooling layers, and fully connected (FC) layers. To capture comprehensive features from the preprocessed sEMG signals, the architecture employs a symmetrical dual-stream configuration: one branch processes four-channel CWT images to capture local time–frequency features, while the parallel branch processes four-channel GADF images to extract temporal-related features.

Within this dual-stream topology, each branch incorporates two identical standard convolutional units for progressive feature extraction. Let $X_{l}\in R^{H\times W\times C}$ denote the output feature map of the l-th convolutional layer. According to the standard definition of a convolutional layer followed by batch normalization (BN) and a rectified linear unit (ReLU) activation function, the forward computation is formulated as

(9) $$X_{l}=\sigma \left(\mathrm{BN}\left(\sum_{k}W_{l}^{k}*X_{l-1}^{k}\right)\right)$$

where ∗ denotes the 2D convolution operation, $W_{l}^{k}$ represents the k-th convolutional kernel tensor at layer l, and $\sigma \left(\cdot \right)=\max\left(0,\;\cdot \right)$ denotes the ReLU activation function. The linear bias is omitted in this stage as it is rendered redundant by the subsequent mean centering of the $\mathrm{BN}\left(\cdot \right)$ operator. The first unit utilizes 16 2D filters (3×3 kernel), followed by a Max-pooling layer with 2×2 filters to reduce spatial dimension. The second convolutional layer increases the feature depth to 32 filters, thereby yielding the intermediate feature map $Z\in R^{H\times W\times C_{in}}$ (where $C_{in}=32$).

**Figure 5:**
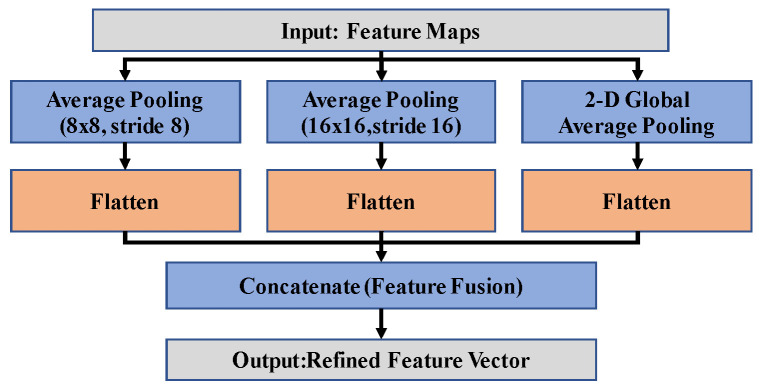
Schematic diagram of an SPP layer incorporating a structure with three parallel average pooling operations.

To effectively aggregate global and local spatial information from the four-channel topologies, the second Max-pooling layer within each branch of the standard DSCNN is substituted with an efficient spatial pyramid pooling (SPP) layer, yielding the dual-stream spatial pyramid pooling CNN (DSSCNN). As depicted in Figure 5, the SPP layer executes three parallel average pooling operations at different spatial scales. Specifically, let $P_{M\times M}\left(\cdot \right)$ denote an average pooling operator with a kernel size and stride of M. The multi-scale feature maps generated by the three parallel paths—parameterized by $P_{8\times 8}\left(\cdot \right)$, $P_{16\times 16}\left(\cdot \right)$, and a 2D global average pooling layer $P_{\mathrm{GAP}}\left(\cdot \right)$—are flattened via a vectorization operator $\mathrm{vec}\left(\cdot \right)$ and concatenated into a single multi-scale spatial vector $Y_{\mathrm{SPP}}$:

(10) $$Y_{\mathrm{SPP}}=\left[\mathrm{vec}\left(P_{8\times 8}\left(Z\right)\right)⊕\mathrm{vec}\left(P_{16\times 16}\left(Z\right)\right)⊕\mathrm{vec}\left(P_{\mathrm{GAP}}\left(Z\right)\right)\right]$$

where ⊕ represents the vector concatenation operation along the feature dimension. Downstream from the SPP layer, the vector $Y_{\mathrm{SPP}}$ undergoes feature refinement through a sub-branch FC layer to generate the branch-specific feature vector $V\in R^{256}$:

(11) $$V={\mathrm{Dropout}}_{0.3}\left(\sigma \left(W_{b}Y_{\mathrm{SPP}}+b_{b}\right)\right)$$

where $W_{b}$ and $b_{b}$ denote the weight matrix and bias vector of the branch FC layer, respectively, and ${\mathrm{Dropout}}_{0.3}\left(\cdot \right)$ represents the regularization operator with a dropout probability of 0.3. The refined feature vectors derived from the CWT branch ($V_{\mathrm{CWT}}$) and the GADF branch ($V_{\mathrm{GADF}}$) are subsequently integrated via a tensor concatenation layer (Cat-Layer) to form the joint multi-stream representation $F_{\mathrm{fusion}}\in R^{512}$:

(12) $$F_{\mathrm{fusion}}=\left[V_{\mathrm{CWT}}⊕V_{\mathrm{GADF}}\right]$$

To prevent overfitting and aggregate high-level abstractions before classification, $F_{\mathrm{fusion}}$ is mapped to a fusion FC layer to yield an intermediate latent representation $H\in R^{128}$:

(13) $$H={\mathrm{Dropout}}_{0.5}\left(\sigma \left(W_{f}F_{\mathrm{fusion}}+b_{f}\right)\right)$$

where $W_{f}$ and $b_{f}$ are the corresponding weight and bias parameters of the fusion block, and the dropout probability is configured to 0.5. Finally, the terminal classification layer maps H into the target label space. The predicted probability distribution $P={\left[p_{1},p_{2},\ldots ,p_{C}\right]}^{T}$ for the gesture classes is given by

(14) $$P=\mathrm{Softmax}\left(W_{c}H+b_{c}\right)$$

To explicitly evaluate individual class likelihoods, the conditional probability $p_{i}$ that the input sEMG pattern belongs to the i-th gesture category is defined as

(15) $$p_{i}=\frac{e^{w_{i}^{T}H+b_{i}}}{\sum_{j=1}^{C}e^{w_{j}^{T}H+b_{j}}}$$

where the classification weight matrix is decomposed into class-specific column vectors as $W_{c}={\left[w_{1},w_{2},\ldots ,w_{C}\right]}^{T}$, and $b_{i}$ denotes the corresponding bias for the i-th class. By replacing conventional rigid pooling with this adaptive multi-scale mathematical mapping, the learnable parameters of the network are reduced from 16.85 MB to 1.40 MB, substantially alleviating training difficulty while optimizing the network’s capacity to encode non-stationary spatial information [29].

### 2.6. Network Optimization and Loss Function

The network parameters are optimized using the Adam algorithm to ensure stable convergence and alleviate redundant gradient oscillations during backpropagation. To evaluate the classification discrepancy, the multi-class cross-entropy loss function is employed as the objective function. Formally, the total loss L over a training mini-batch is defined as [30]:

(16) $$L=-\frac{1}{m}\sum_{k=1}^{m}\sum_{j=1}^{C}y_{k,j}\log\left(p_{k,j}\right)$$

where m denotes the mini-batch size, and C represents the total number of gesture categories. The variable $y_{k,j}\in \{0,1\}$ is the one-hot encoded ground truth indicator for the k-th sample regarding the j-th class, and $p_{k,j}$ is the corresponding normalized conditional probability predicted by the terminal Softmax classifier, satisfying $\sum_{j=1}^{C}p_{k,j}=1$.

## 3. Results

### 3.1. Experimental Parameter Settings

Model training and evaluation were executed on a 64-bit Windows 10 workstation equipped with an Intel Core i9-10900 CPU and an NVIDIA GeForce RTX 2060 GPU. To ensure empirical consistency, all comparative networks were trained under identical hyperparameter configurations on the same sEMG dataset. Specifically, the network parameters were optimized with an initial learning rate of 0.0005, a mini-batch size of 64, and a total training budget of 40 epochs.

### 3.2. Evaluation Criterion

To quantitatively assess and compare the classification performance of the distinct CNN architectures on the dataset, accuracy, precision, recall, and F1-score were adopted as the core evaluation metrics [31,32,33]. These criteria are mathematically defined as follows:

(17) $$\mathrm{Accuracy}=\frac{TP+TN}{TP+TN+FP+FN}$$

(18) $$\mathrm{Precision}=\frac{TP}{TP+FP}$$

(19) $$\mathrm{Recall}=\frac{TP}{TP+FN}$$

(20) $$F1\text{-}\mathrm{Score}=\frac{2\times \mathrm{Precision}\times \mathrm{Recall}}{\mathrm{Precision}+\mathrm{Recall}}$$

where TP, FN, FP, and TN denote the number of true positives, false negatives, false positives, and true negatives, respectively.

### 3.3. Experimental Results

The proposed framework was evaluated on a self-built sEMG gesture dataset. For the intra-subject protocol, samples from each subject were randomized with a fixed seed (seed 42) and split into training and validation subsets at an 8:2 ratio. For additional subject-independent assessment, leave-one-subject-out (LOSO) cross-validation was performed, with data from eight subjects used for training and the remaining subject used for testing in each fold. All baseline models were trained with identical hyperparameter settings to ensure a fair comparison.

To verify the structural advantages of the DSSCNN architecture, its gesture recognition accuracy was compared against the standard DSCNN and a single-branch single-stream CNN (SSCNN). The explicit topological descriptions of the dual-stream DSSCNN and DSCNN models are detailed in Section 2.5, both utilizing CWT and GADF feature images as parallel inputs. Regarding the single-stream baselines, three distinct configurations were implemented: CWT-SSCNN (ingesting only CWT images), GADF-SSCNN (ingesting only GADF images), and CWT-GADF-SSCNN (utilizing a single-stream combination of both modalities). These three SSCNN variants share the identical single-branch and fully connected layer architecture as a single stream of the DSCNN, each comprising two convolutional layers and three fully connected layers under identical parameter settings.

#### 3.3.1. Training Convergence Behavior and Learning Curves

**Figure 6:**
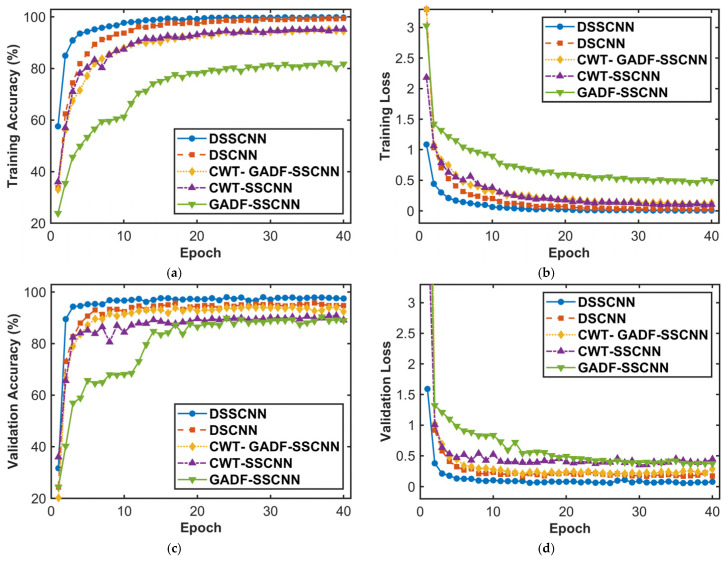
Convergence behavior and different model comparisons based on Subject 1 (S1): (**a**) training accuracy, (**b**) training loss, (**c**) validation accuracy, and (**d**) validation loss.

A total of 3806 feature samples were obtained from five hand gestures performed by Subject 1 (S1) to evaluate the model training performance (Figure 6). As depicted in Figure 6a, the proposed DSSCNN exhibits a rapid upward trend in training accuracy compared to the alternative architectures, reaching a stable convergence state after approximately 15 epochs. Correspondingly, its training loss (Figure 6b) decreases at the highest rate and accelerates toward stable convergence. Furthermore, Figure 6c,d demonstrates that DSSCNN consistently yields the optimal validation accuracy and the lowest validation loss throughout the training phase. These learning curves indicate that the dual-branch configuration, coupled with the multi-scale pooling capability of the integrated SPP layer, significantly accelerates convergence velocity and enhances the model’s generalization capacity over both DSCNN and SSCNN baselines.

#### 3.3.2. Quantitative Evaluation on Single-Subject Dataset

**Figure 7:**
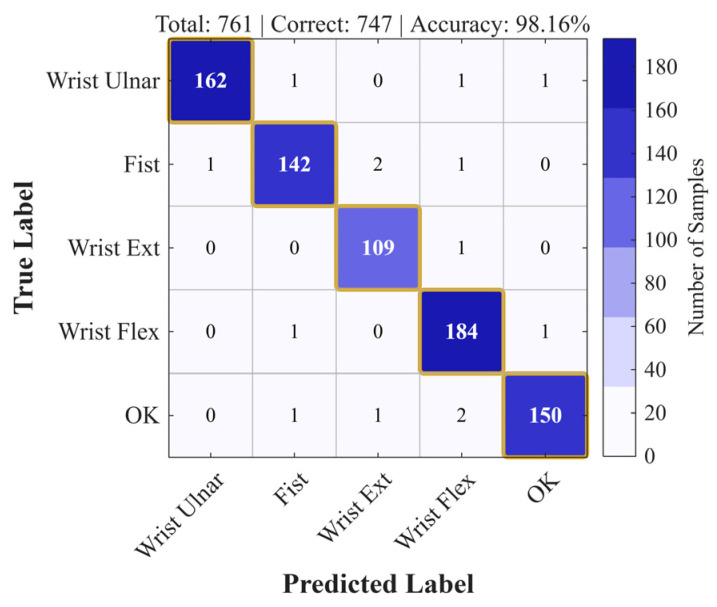
Confusion matrix for DSSCNN-based classification of gestures recognition task on the S1 dataset (N = 761, accuracy = 98.16%).

Figure 7 presents the DSSCNN confusion matrix for gesture recognition on the S1 dataset. The matrix shows strong diagonal dominance across all classes, with per-class accuracies ranging from 97.26% to 99.09%: wrist ulnar deviation, 98.18% (162/165); fist clenching, 97.26% (142/146); wrist extension, 99.09% (109/110); wrist flexion, 98.92% (184/186); and OK gesture, 97.40% (150/154). The overall accuracy was 98.16% (747/761). Most errors occurred between fist clenching and OK gesture, suggesting minor kinematic similarity between these two gestures. With only 14 misclassifications among 761 samples, DSSCNN demonstrates strong discriminative capability across all gesture classes.

**Figure 8:**
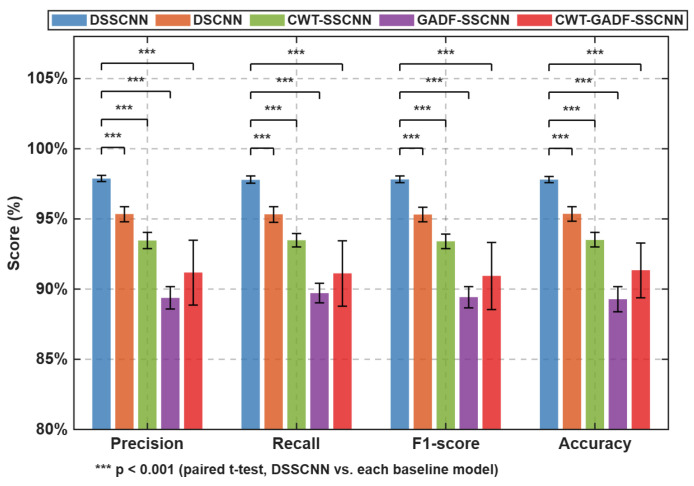
Comparison of precision, recall, and F1-score and accuracy across different network architectures on the S1 dataset.

Figure 8 compares five prediction models on the S1 dataset using accuracy, precision, recall, and F1-score. Under identical image input and training settings, the proposed DSSCNN achieves the highest classification performance across all four evaluation metrics. Its recognition accuracy reached 97.81 ± 0.22%, outperforming the standard DSCNN by 2.45% and the multi-modal single-stream baseline (CWT-GADF-SSCNN) by 4.31%. DSSCNN also outperformed the single-modal CWT-SSCNN and GADF-SSCNN networks, indicating that both dual-domain representation and SPP-based multi-scale aggregation contributed to the observed performance improvement.

Statistical analysis further supported these differences. A Friedman test across the five models showed a significant main effect of model type for all four indicators (Accuracy: χ^2^ = 27.31, *p* < 0.001; Precision: χ^2^ = 26.51, *p* < 0.001; Recall: χ^2^ = 26.51, *p* < 0.001; F1-score: χ^2^ = 26.86, *p* < 0.001). Nemenyi post hoc testing on accuracy showed that DSSCNN significantly outperforms GADF-SSCNN (mean rank difference = 3.86, exceeding the critical difference CD = 2.37 at α = 0.05) and CWT-GADF-SSCNN (mean rank difference = 3.14). Paired *t*-tests further confirm that DSSCNN significantly outperforms all four baselines across the evaluated metrics (all *p* < 0.001, all Cohen’s d > 3.20), as indicated by the *** annotations in Figure 8. Overall, these results provide strong statistical evidence that the proposed DSSCNN framework achieves superior and robust classification performance.

#### 3.3.3. Robustness Evaluation and Comparative Analysis with Existing Studies

**Figure 9:**
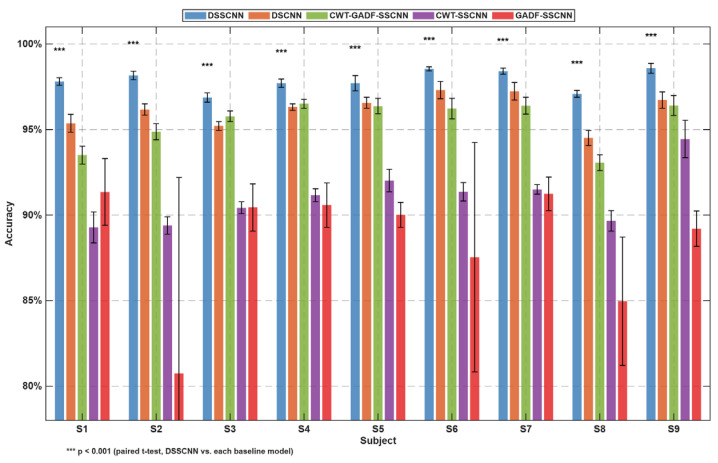
Cross-subject classification accuracy and variability of the proposed DSSCNN and baseline models across 9 subjects.

To evaluate the generalization capability and stability of the proposed SPP-enhanced dual-stream network across individuals, extended validation was conducted across a dataset of nine subjects (Figure 9). The proposed DSSCNN consistently achieves the highest test accuracy across all nine subjects, with an average accuracy of 97.88 ± 0.61%, with the lowest cross-subject variability (CV = 0.62%). In contrast, the single-modal baseline models showed lower and more variable performance (CWT-SSCNN: 91.03 ± 1.62%, CV = 1.78%; GADF-SSCNN: 88.45 ± 3.54%, CV = 4.00%), with GADF-SSCNN displaying significant cross-subject instability. The DSCNN baseline (96.16 ± 0.95%, CV = 0.99%) achieved a moderate improvement, which was higher than the single-stream architectures but lower than DSSCNN.

A Friedman test confirmed a significant main effect of model type across the five models (χ^2^ = 33.51, *p* < 0.001). Nemenyi post hoc analysis showed that DSSCNN significantly outperforms CWT-SSCNN (mean rank difference = 3.22, exceeding CD = 2.09 at α = 0.05) and GADF-SSCNN (mean rank difference = 3.78). Paired *t*-tests further confirmed that DSSCNN outperformed all four baseline models (DSSCNN vs. DSCNN: mean difference = 1.72%, *p* < 0.001, Cohen’s d = 3.19; DSSCNN vs. CWT-GADF-SSCNN: 2.42%, *p* < 0.001, d = 2.02; DSSCNN vs. CWT-SSCNN: 6.85%, *p* < 0.001, d = 4.87; DSSCNN vs. GADF-SSCNN: 9.42%, *p* < 0.001, d = 2.60). These results collectively demonstrate that the proposed DSSCNN framework achieved superior and robust cross-subject generalization performance.

**Figure 10:**
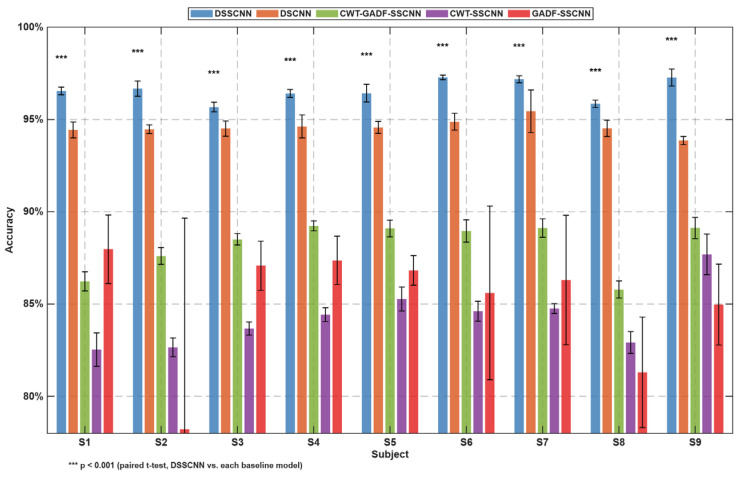
Per-subject recognition accuracy (%) of all five models under LOSO cross-validation.

To further assess the cross-subject generalization capability and reduce potential data leakage with intra-subject random split for time series physiological signals, LOSO cross-validation was also conducted. In each LOSO fold, the model was trained on data from eight subjects and tested on the held-out subject, repeated for all nine subjects. As shown in Figure 10, the proposed DSSCNN achieves an average accuracy of 96.59 ± 0.59% under LOSO evaluation, which was only 1.29% lower than the intra-subject random split result. Notably, The same performance ranking was preserved under the LOSO protocol. Friedman testing showed a significant main effect of model type (χ^2^ = 33.42, *p* < 0.001), and paired *t*-tests confirmed that DSSCNN outperforms all four baseline models (all *p* < 0.001), with large effect sizes (Cohen’s d ranging from 3.02 to 8.90). These results further corroborate the robust cross-subject generalization capability of the proposed framework.

To further demonstrate the efficacy of the proposed DSSCNN framework within the current sEMG gesture recognition research field, the experimental results were compared with representative CNN-based frameworks reported in the literature. As summarized in Table 1, this comparative study encompasses a wide spectrum of input modalities and network architectures, including a deep CNN operating on spectrograms [34]; standard single-stream architectures utilizing raw sEMG sequences [35,36,37]; a multi-scale parallel network driven by multi-modal data fusion [38]; and multi-stream CNN processing multi-feature matrices [6] or CWT maps [22]. Table 1 summarizes the reported classification performance indicators alongside their corresponding input representations, network models, number of participants, and gesture recognition accuracy. Although the sample size of nine subjects is relatively limited, comparable to prior sEMG studies employing 5–10 participants [6,22,36,37,38], the consistently high accuracy and low variability, together with rigorous statistical validation, provide reliable evidence for the robustness of the proposed framework.

#### 3.3.4. Computational Complexity Analysis

To assess the deployability of DSSCNN framework in real-time MCI applications, computational complexity was analyzed across all five models under identical hardware conditions with a batch size of 1. As summarized in Table 2, DSSCNN has a compact model size of 5.62 MB and 1.40 M parameters, representing a 91.67% reduction compared with the standard DSCNN (16.85 M parameters). This reduction is mainly attributed to the SPP layer, which replaces rigid pooling with adaptive multi-scale aggregation and reduces the number of learnable parameters in the fully connected layers. In terms of computational cost, DSSCNN requires only 0.22 GFLOPs per inference, corresponding to an 18.52% reduction over DSCNN (0.27 GFLOPs).

Regarding inference latency, DSSCNN achieved an average inference time of 5.53 ms per sample. Compared to DSCNN baseline (5.01 ms), DSSCNN introduced only a marginal increase of 0.52 ms while improving average recognition accuracy by 1.72%. The single-stream baselines were slightly faster (CWT-GADF-SSCNN: 2.85 ms; CWT-SSCNN: 3.11 ms; GADF-SSCNN: 3.11 ms), but their recognition accuracies were substantially lower (95.46%, 91.03%, and 88.45%, respectively). These findings indicate that DSSCNN provides a favorable trade-off between computational efficiency and recognition accuracy for real-time sEMG-based MCI applications.

### 3.4. Gesture-Based Real-Time Control of an Unmanned Ground Vehicle

**Figure 11:**
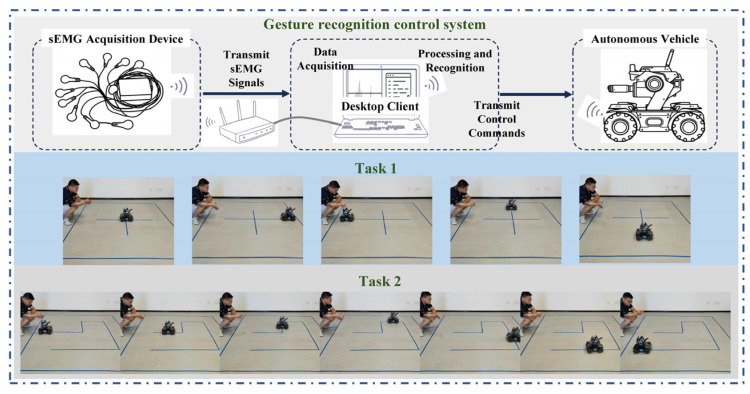
Real-time sEMG-driven control system and experimental verification scenarios (Task 1 and Task 2) on the UGV platform.

To evaluate the practical deployment capability of the proposed DSSCNN framework in real-world scenarios, an online closed-loop control system was established utilizing the RoboMaster EP platform as the unmanned ground vehicle (UGV) execution unit, as shown in Figure 11. The five distinct sEMG gesture patterns validated in the preceding sections—specifically, wrist ulnar deviation, fist clenching, wrist flexion, wrist extension, and the “OK” gesture—were mapped directly into real-time motion commands to control the UGV’s forward, backward, right turn, left turn, and stop maneuvers, respectively.

The operational reliability of the gesture-driven UGV was evaluated via two standard verification scenarios: Task 1 (fixed-point directional movement) and Task 2 (continuous trajectory tracking). In Task 1, the UGV decoded real-time gestures, successfully departing from its initial coordinates toward the designated headings. In Task 2, the UGV smoothly tracked a predefined continuous pathway by processing streaming sEMG sequences. Across both tasks, the system achieved real-time gesture translation and fluid mechanical execution, with control latency remaining within acceptable ranges and decoding failures occurring rarely. The average gesture signal processing and recognition time was approximately 120 ms. This value is comparable to the 153 ms delay reported in a real-time CNN-RNN sEMG recognition system [39]. The total latency of the gesture recognition control system did not exceed 300 ms, which is within the delay range generally considered acceptable for myoelectric control systems [40,41].

## 4. Discussion

The results in Section 3 show that DSSCNN provides strong decoding capability for sEMG-based intent recognition in both offline validation and online closed-loop control. In multi-subject validation, DSSCNN achieved a mean accuracy of 97.88% under the intra-subject random split protocol and 96.59% under LOSO cross-validation. The reduction under LOSO was modest, which demonstrates the model’s effective cross-subject generalization. The single-subject ablation analysis further showed that adding SPP to the DSCNN backbone improved accuracy by 2.45%, reaching 97.81%, while reducing learnable parameters from 16.85 M to 1.40 M. This efficiency supports the real-time UGV control results described in Section 3.4.

The superior decoding capacity and localized stability of DSSCNN are fundamentally rooted in the biophysical mechanisms of motor unit action potential spikes and muscle recruitment dynamics. sEMG signals are derived from the spatial–temporal superposition of motor unit action potential sequences [4], making them inherently non-stationary and highly sensitive to muscle contraction intensity and transient muscle fatigue [1,10]. Conventional rigid pooling operations in typical deep learning models inevitably discard localized high-frequency spatial features when processing non-stationary signal segments, leading to information loss [19,30].

By utilizing a dual-stream architecture, DSSCNN concurrently extracts time–frequency spectrograms via CWT and temporal correlation maps via GADF. The embedded SPP layer further processes these complementary features through three parallel average pooling operations, dynamically aggregating local receptive fields across multiple spatial scales [29,42]. This multi-scale transformation helps reduce feature space distortions caused by minor electrode displacement around the circumferential forearm layout [11,12] and by variation in localized muscle synergy recruitment patterns [8,9]. Consequently, multi-scale spatial pooling preserves discriminative structural features that are difficult to retain in conventional fixed-size convolutional architectures.

This design enables DSSCNN to effectively extracts invariant latent features of gesture patterns, leading to robust cross-subject generalization. The proposed framework achieves 97.88% accuracy under intra-subject random split and 96.59% under the more demanding LOSO protocol, outperforming state-of-the-art models such as the multi-attention CNN reported by Zhang et al. [37] (96.47% accuracy across six gestures). These results confirm that the integration of dual-domain feature encoding and multi-scale spatial pooling provides a structural advantage for sEMG-based intent recognition.

Despite these achievements, several limitations remain. First, the dataset comprised only nine healthy young participants. While this sample size is comparable to prior sEMG studies employing 5–10 subjects [6,22,36,37,38], it remains relatively small for establishing broad population-level generalizability. Second, although the electrode placement was standardized around the proximal forearm, systematic testing for abnormal placement quantification or sensor displacement was not conducted. Third, the validation did not explicitly disentangle the compounding effects of continuous dynamic muscle fatigue and complex limb posture variations, which typically alter sEMG frequency spectra and induce nonlinear baseline drifts during real-time deployments. Furthermore, while the SPP layer successfully compresses the learnable parameters to 1.40 MB, its structural details, such as the number of parallel paths and pooling strides, were assigned empirically, leaving the maximum achievable performance of this multi-scale aggregation layer partially uncharted.

The engineering value of this study lies in its validation of an online hardware-in-the-loop system that operates without expert-level anatomical calibration or localized parameter tuning. On-site UGV testing confirmed real-time gesture translation and smooth mechanical execution, supporting the operational feasibility of the proposed cyber–physical control loop.

Future research will address four limitations. First, multi-center validation should include more diverse participants, including older adults and patients with neuromuscular disorders, to improve population-level generalizability and to connect gesture recognition with broader motion classification applications. Second, systematic sensor displacement and electrode misplacement experiments are needed to quantify robustness under non-ideal deployment conditions. Third, adaptive baseline correction and time–frequency spectral shift detection should be integrated to compensate for muscle fatigue and posture-related drift. Fourth, the discrete gesture classifier should be extended toward continuous state estimation, including mappings from dynamic sEMG signals to multi-joint torques and kinematic parameters [43]. Hybrid CNN–Transformer frameworks may help capture both local activation patterns and long-range temporal dependencies [44,45]. Multi-branch graph neural networks may further model the spatiotemporal coupling between physical electrode channels and localized muscle synergy recruitment patterns [46,47], thereby supporting robust continuous prediction under non-stationary conditions.

## 5. Conclusions

This study proposes a dual-stream spatial pyramid pooling CNN (DSSCNN) to address non-stationarity and cross-subject variability in sEMG-based gesture recognition. By transforming one-dimensional sEMG sequences into complementary CWT time–frequency spectrograms and GADF temporal correlation maps, DSSCNN avoids manual feature engineering and captures informative signal patterns within short windows. The SPP layer further reduces spatial information loss caused by fixed-size pooling and decreases the number of trainable parameters, improving computational flexibility. Compared with the tested single-stream and multi-stream baselines, DSSCNN provided higher classification performance and stable cross-subject behavior. These findings support its potential for non-ideal muscle–computer interfaces, rehabilitation robotics, and human–robot collaboration applications. Further validation with larger and more diverse participant populations is still needed to establish population-level generalizability.

## Figures and Tables

**Table 1:** Performance comparison of the proposed DSSCNN against several state-of-the-art CNN-based sEMG gesture recognition frameworks.

Studies	Input Representation	Network Model	Subjects	Gestures	Accuracy
Duan [34]	Spectrogram	Deep CNN	50	10	94.06%
Asif [35]	Raw sEMG sequences	Standard CNN	18	10	92.00%
Triwiyanto [36]	Raw sEMG sequences	Deep CNN	10	10	93.00%
Gao [38]	Multi-modal data fusion	Multi-scale parallel CNN	6	10	92.45%
Zhang [6]	Sigimg + GADF + MTF	Multi-stream CNN	5	6	82.40%
Tong [22]	CWT maps	Multi-stream CNN	10	9	88.84%
Zhang [37]	Raw sEMG sequences	Multi-attention CNN	10	6	96.47%
The proposed model	CWT + GADF images	Dual-stream SPP-CNN (DSSCNN)	9	5	97.88%

**Table 2:** Computational complexity comparison across five models.

Model	Parameters (M)	Model Size (MB)	FLOPs (G)	Inference Time (ms)
DSSCNN (Proposed)	1.40	5.62	0.22	5.53 ± 0.50
DSCNN	16.85	67.42	0.27	5.01 ± 0.54
CWT-GADF-SSCNN	8.43	33.71	0.28	2.85 ± 0.22
CWT-SSCNN	8.43	33.71	0.22	3.11 ± 0.27
GADF-SSCNN	8.43	33.71	0.22	3.11 ± 0.26

## Data Availability

The data that support the findings of this study are available from the corresponding author upon reasonable request.
